# Standardized uptake values of ^99m^Tc-MDP in normal vertebrae assessed using quantitative SPECT/CT for differentiation diagnosis of benign and malignant bone lesions

**DOI:** 10.1186/s12880-021-00569-5

**Published:** 2021-02-27

**Authors:** Na Qi, Qingyuan Meng, Zhiwen You, Huiqian Chen, Yi Shou, Jun Zhao

**Affiliations:** Department of Nuclear Medicine, Shanghai East Hospital, Tongji University School of Medicine, No. 150 Jimo Rd, Shanghai, 200120 China

**Keywords:** SUV, SPECT/CT, Bone scintigraphy, Vertebra, Quantification

## Abstract

**Background:**

Quantitative bone SPECT/CT is useful for disease follow up and inter-patient comparison. For bone metastatic malignant lesions, spine is the most commonly invaded site. However, Quantitative studies with large sample size investigating all the segments of normal cervical, thoracic and lumbar vertebrae are seldom reported. This study was to evaluate the quantitative tomography of normal vertebrae using ^99m^Tc-MDP with SPECT/CT to investigate the feasibility of standardized uptake value (SUV) for differential diagnosis of benign and malignant bone lesions.

**Methods:**

A retrospective study was carried out involving 221 patients (116 males and 105 females) who underwent SPECT/CT scan using ^99m^Tc-MDP. The maximum SUV (SUV_max_), mean SUV (SUV_mean_) and CT values (Hounsfield Unit, HU) of 2416 normal vertebrae bodies, 157 benign bone lesions and 118 malignant bone metastasis foci were obtained. The correlations between SUV_max_ of normal vertebrae and CT values of normal vertebrae, age, height, weight, BMI of patients were analyzed. Statistical analysis was performed with data of normal, benign and malignant groups corresponding to same sites and gender.

**Results:**

The SUV_max_ and SUV_mean_ of normal vertebrae in males were markedly higher than those in females (*P* < 0.0009). The SUV_max_ of each normal vertebral segment showed a strong negative correlation with CT values in both males and females (r = − 0.89 and − 0.92, respectively; *P* < 0.0009). The SUV_max_ of normal vertebrae also showed significant correlation with weight, height, and BMI in males (r = 0.4, *P* < 0.0009; r = 0.28, *P* = 0.005; r = 0.22, *P* = 0.026), and significant correlation with weight and BMI in females (r = 0.32, *P* = 0.009; r = 0.23, *P* = 0.031). The SUV_max_ of normal group, benign bone lesion group and malignant bone metastasis foci group showed statistical differences in both males and females.

**Conclusion:**

Our study evaluated SUV_max_ and SUV_mean_ of normal vertebrae, benign bone lesion and malignant bone metastasis foci with a large sample population. Preliminary results proved the potential value of SUV_max_ in differentiation benign and malignant bone lesions. The results may provide a quantitative reference for clinical diagnosis and the evaluation of therapeutic response in vertebral lesions.

**Supplementary Information:**

The online version contains supplementary material available at 10.1186/s12880-021-00569-5.

## Background

Radionuclide bone imaging is the most frequently used imaging technology in nuclear medicine, accounting for about 60.3% in hybrid single photon emission computed tomography/computed tomography (SPECT/CT) examination per year in China [1]. Up to now, the differentiation between lesions and normal bone tissue was mainly based on visual diagnosis, while quantitative analysis has not been well applied in clinic. Quantitative positron emission tomography (PET) bone imaging based on fluorine-18-sodium fluoride (^18^F-NaF) is considered to have potential clinical value. However, ^18^F-NaF PET/CT is quite expensive with limited availability [2]. The development of SPECT/CT technology has enabled quantitative assessments of bone imaging using Technetium-99m methylene diphosphonate (^99m^Tc-MDP), a prevailing used SPECT tracer for bone imaging. Compared to ^18^F-NaF, ^99m^Tc-MDP was more frequently used for the bone imaging in clinic [3]. Beck et al. [4] found that, for SPECT/CT bone imaging, quantitative analysis showed high agreement among the observers. Arvola et al. [5] proved that standardized uptake value (SUV) obtained from SPECT images of bone metastases of breast and prostate cancer were significantly correlated with SUV obtained from PET images. These findings indicated the feasibility of SPECT quantification using SUV in clinic.

Bone tissue uptake of ^99m^Tc-MDP is proportional to blood flow and osteoblastic activity [6]. Hence, bones at different sites can have different normal SUVs. Bone metastasis is a common complication of cancer [7], and spine is the most commonly invaded site [8]. Therefore, establishing the SUV range of normal vertebrae is of great value in clinical practice. Kaneta et al. [9] demonstrated that SUV_max_ had the lowest variance coefficient, indicating SUV_max_ was a suitable quantitative indicator in bone imaging.

To our best knowledge, present quantitative studies [9–11] only conducted with small sample sizes evaluated SUV of SPECT imaging of partial normal vertebrae (mostly are lumbar vertebrae). Quantitative studies with large sample size investigating all the segments of normal cervical, thoracic and lumbar vertebrae are seldom reported. xSPECT Quant using a 3% National Institute of Standards and Technology (NIST) traceable calibration for system sensitivity calibration and cross calibration of the dose calibrator, enable standardization of quantitative SPECT based on OSCGM reconstruction algorithm [12]. The aim of this study was to obtain the SUV_max_ and SUV_mean_ in normal vertebrae using ^99m^Tc-MDP-SPECT/CT and to investigate the clinical value of quantitative SPECT/CT in differentiation of benign bone lesions and malignant vertebral metastasis.

## Methods

### Patients

Retrospective analysis was performed on patients who underwent SPECT/CT scan in Shanghai East Hospital from August 2016 to October 2019, and all patients or family members signed informed consent for examination. This retrospective study was approved by institutional review board of Shanghai East Hospital. The following are the patients’ inclusion criteria: no history of primary bone tumor; no history of renal insufficiency; no history of hormone, endocrine therapy, chemotherapy and other treatments affecting bone metabolism; access to the information of patients’ height, weight, measured injection activity (full needle and empty needle), time of tracer injection, and time of SPECT/CT acquisition.

### SPECT/CT acquisition

All subjects were injected with 19–22 MBq/kg ^99m^Tc-MDP. Whole-body planar imaging and tomographic imaging were performed at about 3 h post injection, which took approximately 40 min. Patients were scanned on SPECT/CT (Siemens Symbia Intevo, Erlangen, Germany), a low energy high resolution collimator with a single probe rotation 30 projections over 180° with 20 s acquisition time per view, 256 × 256 matrix, pixel size 2.4 × 2.4 mm^2^, 2.4 mm thickness. Low-dose CT scan was performed at 130 kV and 10 valid mAs. CT data was reconstructed using a smooth attenuation-correction kernel B31s with 3 mm slice thickness and a sharp bone kernel B50s with 5 mm slice thickness. SPECT reconstruction was performed based on the B31s CT attenuation map of ordered subsets conjugate gradient (OSCG) enhanced with 2 subsets and 28 iterations without post-smoothing, which generate SPECT data allowing SUV based on body weight (SUV_bw_) quantification and measurement of SUV_max_ and SUV_mean_ using xSPECT reconstruction algorithm (xSPECT/CT, Siemens Symbia Intevo).

### Image analysis

**Fig. 1:**
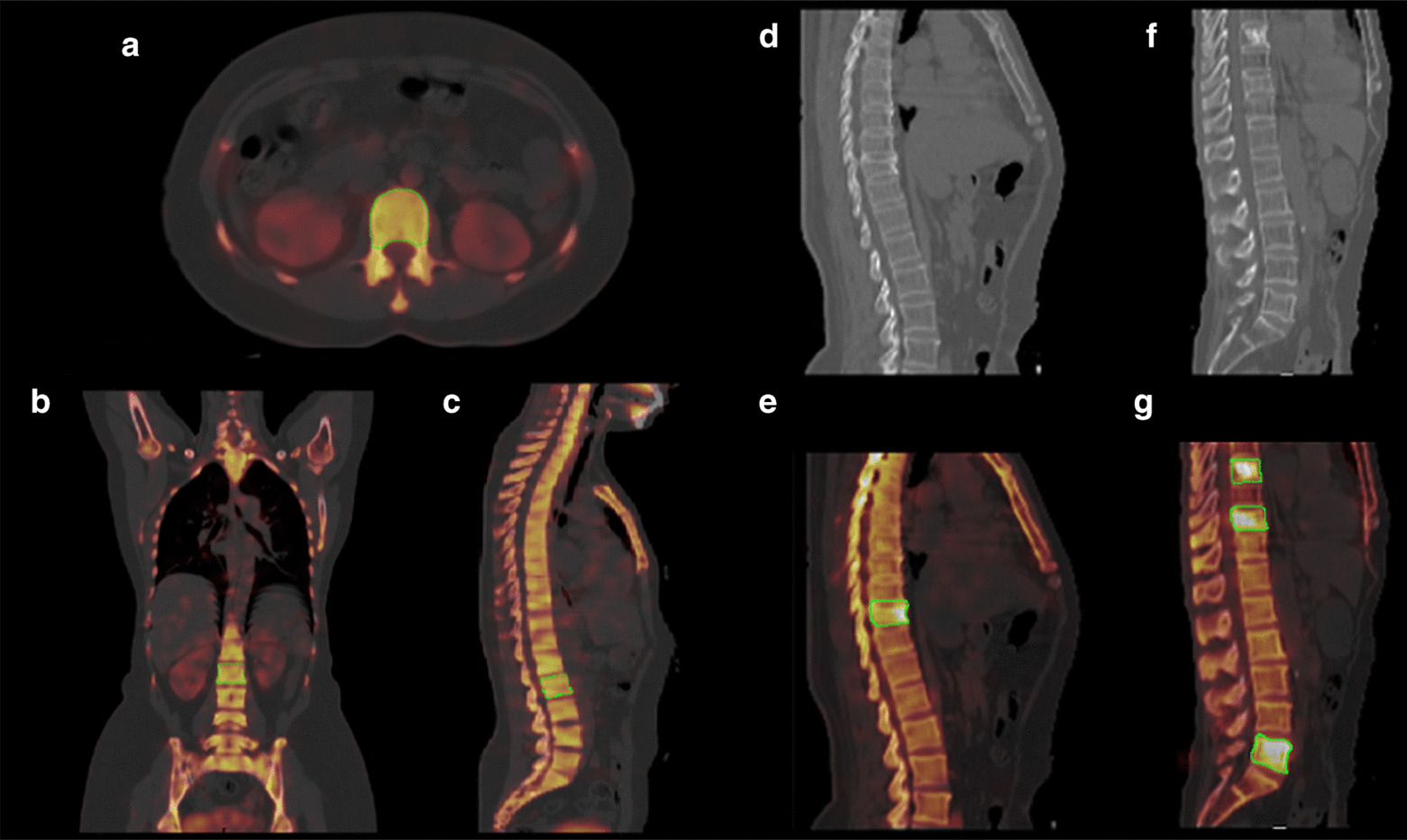
Representative SPECT/CT images of normal vertebrae, benign and malignant bone lesions. Transaxial (**a**), coronal (**b**) and sagittal (**c**) SPECT/CT images of a patient with normal thoracic and lumbar regions. The ellipses depicted the VOI of L2(using Siemens 3D Isocontour), SUV_max_ = 6.75; Sagittal CT (**d**) and SPECT/CT (**e**) images of a patient with T10 compression fracture (SUV_max_ = 13.06); Sagittal CT (**f**) and SPECT/CT (**g**) images showed a patient who had surgery of prostate cancer 5 years ago with T8, T10, and L5 bone metastases (SUV_max_ are 19.76, 18.27, 19.69 respectively)

Two experienced nuclear medicine physicians interpreted the ^99m^Tc-MDP planar and SPECT/CT images independently. Discordant results reached consensus with joint reading. Normal vertebrae, benign bone lesion and malignant bone metastasis foci were categorized based on the image interpretation results with follow-up or other diagnostic imaging inspect such as CT or MRI. Volume of interest (VOI, Siemens 3D Isocontour) were drew on sagittal position with SUV automatically calculated. For normal vertebrae, both cortical bone and trabecular bone were included within VOIs and SUV_max_, SUV_mean_ and CT values were recorded. For lesions, elliptical VOIs were drew over the hottest area and SUV_max_ were obtained. Figure 1 showed the representative coronal, sagittal, and transversal images with VOIs of normal vertebrae and bone lesions.

### Statistical analysis

Shapiro–Wilk normality test was used to analyze the data distribution. Data were expressed as mean ± standard deviation. Data of male and female groups were tested by independent sample T test. Pearson correlation analysis was performed between SUV_max_ and CT value, height, weight, BMI, age. Statistical analysis was performed using SPSS 23.0 statistical software. SUV_max_ of normal vertebrae, benign lesion and malignant bone metastasis foci were compared corresponding to same site and sex. *P* < 0.05 was considered statistically significant.

## Results

### Patient data

A total of 221 patients were included in this study. The collected data and statistical analysis of male and female patients were listed in Table 1. The detailed inclusion number, CT value, SUV_max_ and SUV_mean_ of normal vertebrae in male and female patients were shown in Additional file 1 and 2, respectively.

**Table 1:** Patient demographics

Parameters		Male (n = 116)			Female (n = 105)		
		Range	Mean	SD	Range	Mean	SD
Age (years)		28–89	66.3	10.8	29–89	62. 8	± 11.5
Weight (kg)		35–85	62.8	11	37–90	58.5	10.2
Height (cm)		150–181	168.4	6.3	145–173	148.9	5.4
BMI (kg/m^2^)		4.9–11.0	7.9	1.3	3.1–11.1	7.1	1.5
Parameters		mean	SD	CoV	mean	SD	CoV
Normal vertebrae (n = 2416)	CT values (HU)	224.9	100	0.44	224.2	93.2	0.42
	SUVmax	7.87	1.57	0.19	6.97	1.63	0.23
	SUVmean	4.97	1.02	0.2	4.59	1.02	0.22
Benign bone lesion (n = 157)	Thoracic SUVmax (n = 51)	10.24	1.50	0.15	10.67	1.61	0.15
	Lumbar SUVmax (n = 106)	14.10	4.58	0.32	13.72	4.28	0.31
Malignant bone metastasis foci (n = 118)	Thoracic SUVmax (n = 56)	20.37	12.18	0.60	15.39	6.97	0.45
	Lumbar SUVmax (n = 62)	27.04	13.91	0.51	18.12	6.41	0.35

CoV: coefficient of variation

### Comparison of SUV data of normal vertebrae between male and female patients

**Fig. 2:**
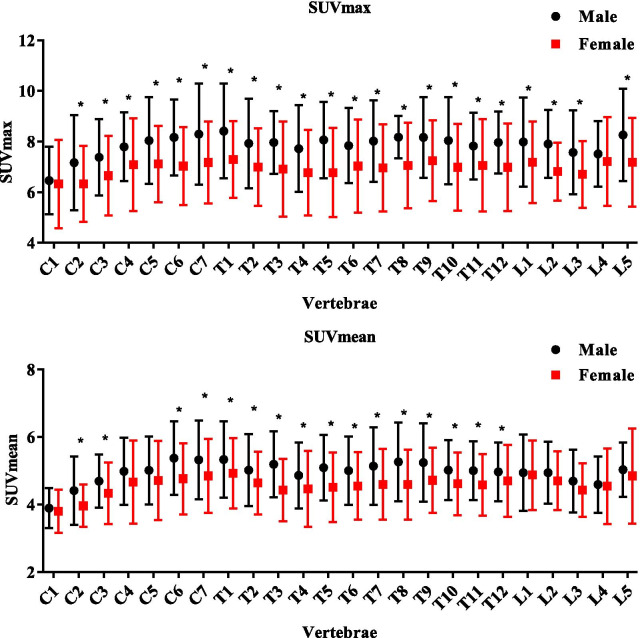
SUVmax and SUVmean of normal vertebrae, *P < 0.05

**Fig. 3:**
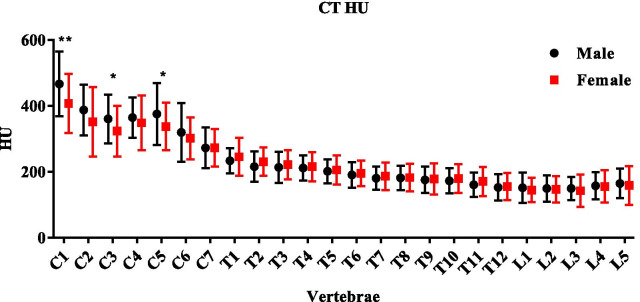
CT value (HU) of normal vertebra

SUV_max_ and SUV_mean_ of each normal vertebra were compared between male and female patients using Paired t-test (Fig. 2). Significant statistical differences were observed in SUV_max_ of almost all the vertebrae (91.6%) except C1 and L4 vertebrae. And for SUV_mean_, more than half of the vertebrae (62.5%) showed significant differences between male and female patients, among which no lumbar vertebra was found with significant differences between two groups. The specific *P* values of each vertebral segment were listed in Additional file 3. We summarized the range, mean and standardized deviation value of SUV_max_ in cervical, thoracic, and lumbar vertebrae of male and female patients respectively in Table 2 as a normal reference. It demonstrated that the SUV_max_ of normal cervical, thoracic, lumbar vertebrae in male patients were significantly higher than that in female (*P* < 0.0009). SUV_max_ of each vertebral region (cervical, thoracic, lumbar vertebrae) also showed significant differences in male patients (Additional file 4). In the meanwhile, CT values did not show significant differences between male and female patients (Fig. 3).

**Table 2:** SUVmax of normal cervical, thoracic, lumbar vertebrae in male and female patients

Vertebrae	SUVmax (range, mean ± SD)		T-test
	Male	Female	*P*
CERVICAL	3.29–12.69, 7.66 ± 1.74	2.47–10.89, 6.85 ± 1.64	< 0.0009
THORACIC	4.08–12.61, 8.01 ± 1.52	2.50–11.69, 7.01 ± 1.68	< 0.0009
LUMBAR	3.91–11.83, 7.75 ± 1.46	3.19–11.27, 7.04 ± 1.47	< 0.0009

### Correlation analysis of data from normal group

Correlations between SUV_max_ of normal vertebrae and CT value (HU), age, weight, height, BMI were analyzed separately (Table 3). A strong negative correlation was found between SUV_max_ and CT value in both male (r = − 0.89; *P* < 0.0009) and female (r = − 0.91; *P* < 0.0009) groups. For male patients, correlations of vertebral SUV_max_ with height, weight, BMI were found to be positive (r = 0.28, *P* = 0.005; r = 0.4, *P* < 0.0009; r = 0.22, *P* = 0.026), while no significant correlation was observed between vertebral SUV_max_ and age. For female patients, vertebral SUV_max_ had no significant correlation with age and height but had positive correlations with body weight and BMI (r = 0.32, *P* = 0.009; r = 0.23, *P* = 0.031).

**Table 3:** Correlations between SUVmax and CT values of normal vertebrae, age, height, weight and BMI in male and female groups

	Male		Female	
	r	*P*	r	*P*
CT value	− 0.89	< 0.0009*	− 0.92	< 0.0009*
Age	− 0.45	0.656	0.23	0.069
Height	0.28	0.005*	0.22	0.075
Weight	0.4	< 0.0009*	0.32	0.009*
BMI	0.22	0.026*	0.23	0.031*

r represents the correlation coefficient of Pearson correlation analysis, *P* represents the significance, *P < 0.05

### Comparison of SUV_max_ in normal vertebrae, benign bone lesion and malignant bone metastasis foci

**Fig. 4:**
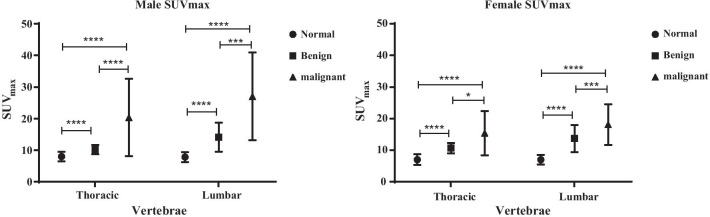
SUVmax of normal vertebrae, benign bone lesion and malignant bone metastasis foci, *P < 0.05

SUV_max_ of thoracic and lumbar vertebrae in benign and malignant groups were listed in Table 1. As shown in Fig. 4, when comparing SUV_max_ of normal, benign and malignant groups, statistical differences were shown in each vertebral region of both male and female patients.

## Discussion

In this study, we assessed the SUV of ^99m^Tc-MDP in normal vertebrae, benign bone lesion and malignant bone metastasis foci using quantitative SPECT/CT in 221 patients. For normal vertebrae, we evaluated the SUV_max_, SUV_mean_ and CT value (HU) of all the 24 vertebral segments in male and female patients. It showed that SUV_max_ in male patients were markedly higher than those in females. In addition, we found that SUV_max_ of three vertebral regions in male patients also showed statistically differences. When comparing SUV_max_ in different vertebral regions between male and female patients, SUV_max_ were proved to be significantly different between male and female patients in cervical, thoracic and lumber vertebrae. This reminds us that to establish a quantitative diagnostic reference for differentiating vertebral lesions, lesions should be categorized based on gender and vertebral regions to compare the SUV_max_.

Cachovan et al. [10] used SPECT/CT bone quantification to obtain the mean SUV_max_ of Tc-99m diphosphono-sponge propanedi-carboxylic acid (^99m^Tc-DPD) of L3-5 vertebral trabecular bone in 50 females (mean ± SD = 5.91 ± 1.54). In our study, the mean SUV_max_ of lumbar in female participants was 7.04 ± 1.47, which was slightly higher than the value obtained by Cachovan et al. The small different results may due to the different tracer kinetics. Furthermore, in our study, the VOI contained the cortical bone with high bone salt metabolism which could lead to the increased SUV_max_. The SUV_max_ of normal vertebra in our study was similar to the previously reported studies which also included bone cortex in their VOIs [11, 13–18]. It is well known that bone lesions including tumors, inflammation and other diseases often ended up with cortical hyperplasia [18, 19]. Therefore, we suggest the inclusion of cortical bone within the VOI for quantitative analysis.

Besides, we analyzed the correlation between SUV_max_ of normal vertebrae and CT values (HU), age, height, weight, BMI in male and female patients. It comes out that SUV_max_ of normal vertebrae showed a strong negative correlation with CT values in both men and women. The SUV_max_ of normal vertebrae also showed significant correlation with weight, height and BMI in male patients, and significant correlation with weight and BMI in female patients.

Hounsfield Unit (HU) is a commonly used measurement index in CT images that indicates the X-ray attenuation degree in tissue (also known as bone density). Bone mineral density (BMD) obtained through dual energy X-ray absorptiometry (DEXA) is the gold standard for the measurement of BMD in clinic [20]. There is still controversy about the relationship between HU and SUV. Previous studies demonstrated a significant positive correlation between HU and BMD [10, 11] [21–24], which was opposite to our findings. To figure out the reason leading to this controversial result, we found that previous studies only analyzed relationship in the lumbar vertebral region. As shown in Fig. 3, HU showed a decreasing trend from cervical to lumbar vertebra. In the meanwhile, SUV_max_ didn’t show clear changing trend. SUV_max_ of ^99m^Tc-MDP in bone is often associated with blood supply and osteoblastic activity [6]. The blood supply of the lumbar artery from the abdominal aorta is richer than that from the vertebral artery [22]. Subjected by gravity and effected by weight, pressure increased from cervical to lumbar leading to more osteoblastic activity in lumber vertebra [9]. And due to the anatomical structure, pressure load of lumbar is predominantly static, while cervical is mainly dynamic. In addition, the cervical spine also needs to move in all three planes. The distribution of tension lines in different directions leads to a denser trabecular structure in the cervical [25]. Hence BMD of lumbar vertebra was lower than the cervical vertebra resulting in a lower CT HU value, while richer blood supply in lumbar enhanced the tracer uptake. Besides, the age of the subjects, imaging parameters and reconstruction algorithm in different experiments may also lead to different results. Our results were consistent with some other studies [23, 24]. Israel et al. [23] found that the ^99m^Tc-MDP uptake in bone cortex of osteoporotic women was higher than that of non-osteoporotic women, suggesting that the bone loss in osteoporosis patients may increase bone conversion, leading to the increase of the bone cortex uptake of ^99m^Tc-MDP. Fogelman et al. [24] performed SPECT on young women after ovariectomy and found a negative correlation between MDP distribution and BMD. As Table 1 showed, the mean age in our study was 66.3 of male and 62.8 of female. With an increasing possibility of calcium loss, the negative correlation between SUV_max_ and CT value was observed. It suggests, using hybrid imaging of SPECT/CT, combining SUV_max_ and CT value (HU) could be used as a potential biological indicator for the evaluation of osteoporosis, and establishing the SUV_max_ and HU of normal vertebral bodies should be taken into consideration. But the mechanism underlying this correlation still need further investigation.

The relationship of SUV and height and weight also showed opposite results with previous studies [7, 9, 11]. It has been reported that SUV_max_ of vertebra was independent with height. Maybe the limited sample size in previous studies lead to the controversial results. With a large sample size, our results showed that SUV_max_ was positively correlated with the height, weight in men and positively correlated with the weight in women which further validated the hypothesis proposed by Kaneta et al. [9] that the increased pressure leads to more blood supply, thus resulting in the increasing of SUV_max_. We also found that SUV_max_ was positively correlated with BMI. To our knowledge, few studies have reported such a relationship. A PET/CT study using ^18^F-NaF assessed the effects of BMI on knee joint inflammation and found a positive correlation between ^18^F-NaF uptake in knee joint and BMI, which was similar with our findings [26]. It is also potentially caused by the increased mechanical loading which increased the blood supply and bone turnover, and as a result an increasing tracer uptake. But further research is required to clarify these findings. In our study, the SUV_max_ and SUV_mean_ were significantly higher in men than those in women. This might also due to the height of women in our study was generally lower than men. Since the number of patients in each age group was not evenly distributed by height, we did not find a correlation between SUV_max_ and age.

With the established reference of SUV_max_ in normal vertebrae (Table 2), SUV_max_ of bone lesions were compared with normal reference to verify the differentiating diagnostic value of quantitative SPECT/CT in bone scanning. Our results demonstrated that SUV_max_ of normal vertebrae, benign bone lesion and malignant bone metastasis foci were significantly different from each other in thoracic and lumbar regions of male and female patients. The results were consistent with previous studies [16, 17], which verified the differentiating diagnostic value of SUV_max_ in bone lesions using SPECT/CT. Different from previous studies, based on our findings in normal vertebrae, data comparison was performed on lesions in different gender and vertebral region groups. Hence, we suggest quantitative diagnose of bone lesions using SPECT/CT should take gender and vertebral regions into consideration. These results also remind us that quantitative SPECT/CT may be of great value in therapy monitoring.

Our study has some limitations. Firstly, although age distribution was wide, the uneven distribution of research object number of each age group could make it hard to reflect the SUV_max_ of all ages. Secondly, the SUV_max_ acquired in our study was based on body weight. Since the ^99m^Tc-MDP uptake mainly exists in bone, the standardization of bone volume can improve the accuracy of quantification [27]. This indicated that bone volume should be included in future studies. In addition, the quantitative accuracy of bone imaging is also affected by the reconstruction parameters. Previous studies have shown that quantitative values increase with higher number of iterations [28]. Therefore, in future studies, we will further expand the sample size and stratify experimental subjects according to age, height and BMI. We will further optimize reconstruction parameters such as increasing the number of iterations to obtain more accurate bone quantitative standard values. Although CT value (HU) was evaluated in normal vertebrae, we didn’t investigate the diagnostic value of combination of SUV_max_ and HU in differentiating bone lesions. This is a promising topic in quantitative SPECT/CT, we will explore its potential diagnostic value in the future.

## Conclusion

In this study, SUV_max_ and SUV_mean_ of normal vertebrae were evaluated. SUV_max_ of male patients was significantly higher than female patients in different vertebral regions. Quantitative SPECT/CT using ^99m^Tc-MDP was demonstrated to have the diagnostic value in differentiation bone lesions of vertebrae. SUV_max_ comparison should be performed considering different gender and vertebral region.

## Supplementary Information

**Additional file 1**. Included number, CT value, SUV_max_ and SUV_mean_ of normal vertebrae in male participants.

**Additional file 2**. Included number, CT value, SUV_max_ and SUV_mean_ of normal vertebrae in female participants.

**Additional file 3**. Differences of SUV_max_ and SUV_mean_ between male and female participants in each vertebra.

**Additional file 4**. SUV_max_ of normal vertebrae.

## Data Availability

All data generated or analyzed during this study are included in this published article [and its supplementary information files].

## Abbreviations

^99m^Tc-MDP: Technetium-99m methylene diphosphonate
^99m^Tc-DPD: Tc-99m diphosphono-sponge propanedi-carboxylic acid
^18^F-NaF: Fluorine-18-sodium fluoride
BMD: Bone mineral density
DEXA: Dual energy X-ray absorptiometry
HU: Hounsfield Unit
OSCG: Ordered subsets conjugate gradient
PET: Positron emission tomography
SPECT/CT: Single photon emission computed tomography/computed tomography
SUV: Standardized uptake value
SUV_max_: Maximum standardized uptake value
SUV_mean_: Mean standardized uptake value
SUV_bw_: Standardized uptake value based on body weight
VOI: Volume of interest
