# Supplementary material for: Standardized uptake values of 99mTc-MDP in normal vertebrae assessed using quantitative SPECT/CT for differentiation diagnosis of benign and malignant bone lesions
Source: BMC Med Imaging. 2021 Feb 27;21:39. doi: 10.1186/s12880-021-00569-5 (PMC7913396; doi:10.1186/s12880-021-00569-5)
Supplement: Supplementary file 1 — Additional file 1. Included number, CT value, SUVmax and SUVmean of normal vertebrae in male participants. [file 12880_2021_569_MOESM1_ESM.pptx]

## Slide 1
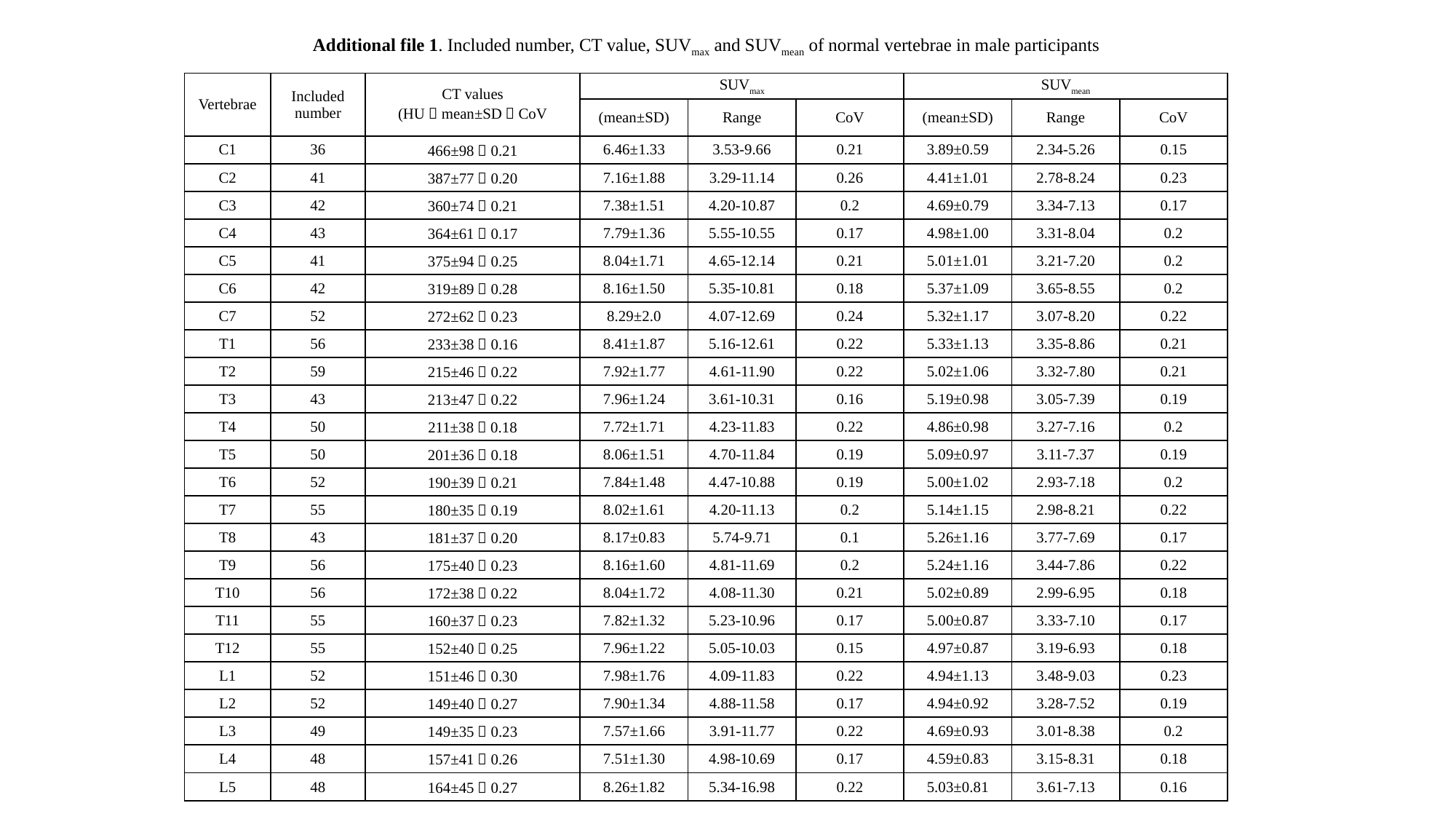

Additional file 1. Included number, CT value, SUVmax and SUVmean of normal vertebrae in male participants
| Vertebrae | Included number | CT values (HU）mean±SD，CoV | SUVmax | | | SUVmean | | |
| --- | --- | --- | --- | --- | --- | --- | --- | --- |
| | | | (mean±SD) | Range | CoV | (mean±SD) | Range | CoV |
| C1 | 36 | 466±98，0.21 | 6.46±1.33 | 3.53-9.66 | 0.21 | 3.89±0.59 | 2.34-5.26 | 0.15 |
| C2 | 41 | 387±77，0.20 | 7.16±1.88 | 3.29-11.14 | 0.26 | 4.41±1.01 | 2.78-8.24 | 0.23 |
| C3 | 42 | 360±74，0.21 | 7.38±1.51 | 4.20-10.87 | 0.2 | 4.69±0.79 | 3.34-7.13 | 0.17 |
| C4 | 43 | 364±61，0.17 | 7.79±1.36 | 5.55-10.55 | 0.17 | 4.98±1.00 | 3.31-8.04 | 0.2 |
| C5 | 41 | 375±94，0.25 | 8.04±1.71 | 4.65-12.14 | 0.21 | 5.01±1.01 | 3.21-7.20 | 0.2 |
| C6 | 42 | 319±89，0.28 | 8.16±1.50 | 5.35-10.81 | 0.18 | 5.37±1.09 | 3.65-8.55 | 0.2 |
| C7 | 52 | 272±62，0.23 | 8.29±2.0 | 4.07-12.69 | 0.24 | 5.32±1.17 | 3.07-8.20 | 0.22 |
| T1 | 56 | 233±38，0.16 | 8.41±1.87 | 5.16-12.61 | 0.22 | 5.33±1.13 | 3.35-8.86 | 0.21 |
| T2 | 59 | 215±46，0.22 | 7.92±1.77 | 4.61-11.90 | 0.22 | 5.02±1.06 | 3.32-7.80 | 0.21 |
| T3 | 43 | 213±47，0.22 | 7.96±1.24 | 3.61-10.31 | 0.16 | 5.19±0.98 | 3.05-7.39 | 0.19 |
| T4 | 50 | 211±38，0.18 | 7.72±1.71 | 4.23-11.83 | 0.22 | 4.86±0.98 | 3.27-7.16 | 0.2 |
| T5 | 50 | 201±36，0.18 | 8.06±1.51 | 4.70-11.84 | 0.19 | 5.09±0.97 | 3.11-7.37 | 0.19 |
| T6 | 52 | 190±39，0.21 | 7.84±1.48 | 4.47-10.88 | 0.19 | 5.00±1.02 | 2.93-7.18 | 0.2 |
| T7 | 55 | 180±35，0.19 | 8.02±1.61 | 4.20-11.13 | 0.2 | 5.14±1.15 | 2.98-8.21 | 0.22 |
| T8 | 43 | 181±37，0.20 | 8.17±0.83 | 5.74-9.71 | 0.1 | 5.26±1.16 | 3.77-7.69 | 0.17 |
| T9 | 56 | 175±40，0.23 | 8.16±1.60 | 4.81-11.69 | 0.2 | 5.24±1.16 | 3.44-7.86 | 0.22 |
| T10 | 56 | 172±38，0.22 | 8.04±1.72 | 4.08-11.30 | 0.21 | 5.02±0.89 | 2.99-6.95 | 0.18 |
| T11 | 55 | 160±37，0.23 | 7.82±1.32 | 5.23-10.96 | 0.17 | 5.00±0.87 | 3.33-7.10 | 0.17 |
| T12 | 55 | 152±40，0.25 | 7.96±1.22 | 5.05-10.03 | 0.15 | 4.97±0.87 | 3.19-6.93 | 0.18 |
| L1 | 52 | 151±46，0.30 | 7.98±1.76 | 4.09-11.83 | 0.22 | 4.94±1.13 | 3.48-9.03 | 0.23 |
| L2 | 52 | 149±40，0.27 | 7.90±1.34 | 4.88-11.58 | 0.17 | 4.94±0.92 | 3.28-7.52 | 0.19 |
| L3 | 49 | 149±35，0.23 | 7.57±1.66 | 3.91-11.77 | 0.22 | 4.69±0.93 | 3.01-8.38 | 0.2 |
| L4 | 48 | 157±41，0.26 | 7.51±1.30 | 4.98-10.69 | 0.17 | 4.59±0.83 | 3.15-8.31 | 0.18 |
| L5 | 48 | 164±45，0.27 | 8.26±1.82 | 5.34-16.98 | 0.22 | 5.03±0.81 | 3.61-7.13 | 0.16 |
