# Supplementary material for: Standardized uptake values of 99mTc-MDP in normal vertebrae assessed using quantitative SPECT/CT for differentiation diagnosis of benign and malignant bone lesions
Source: BMC Med Imaging. 2021 Feb 27;21:39. doi: 10.1186/s12880-021-00569-5 (PMC7913396; doi:10.1186/s12880-021-00569-5)
Supplement: Supplementary file 2 — Additional file 2. Included number, CT value, SUVmax and SUVmean of normal vertebrae in female participants. [file 12880_2021_569_MOESM2_ESM.pptx]

## Slide 1
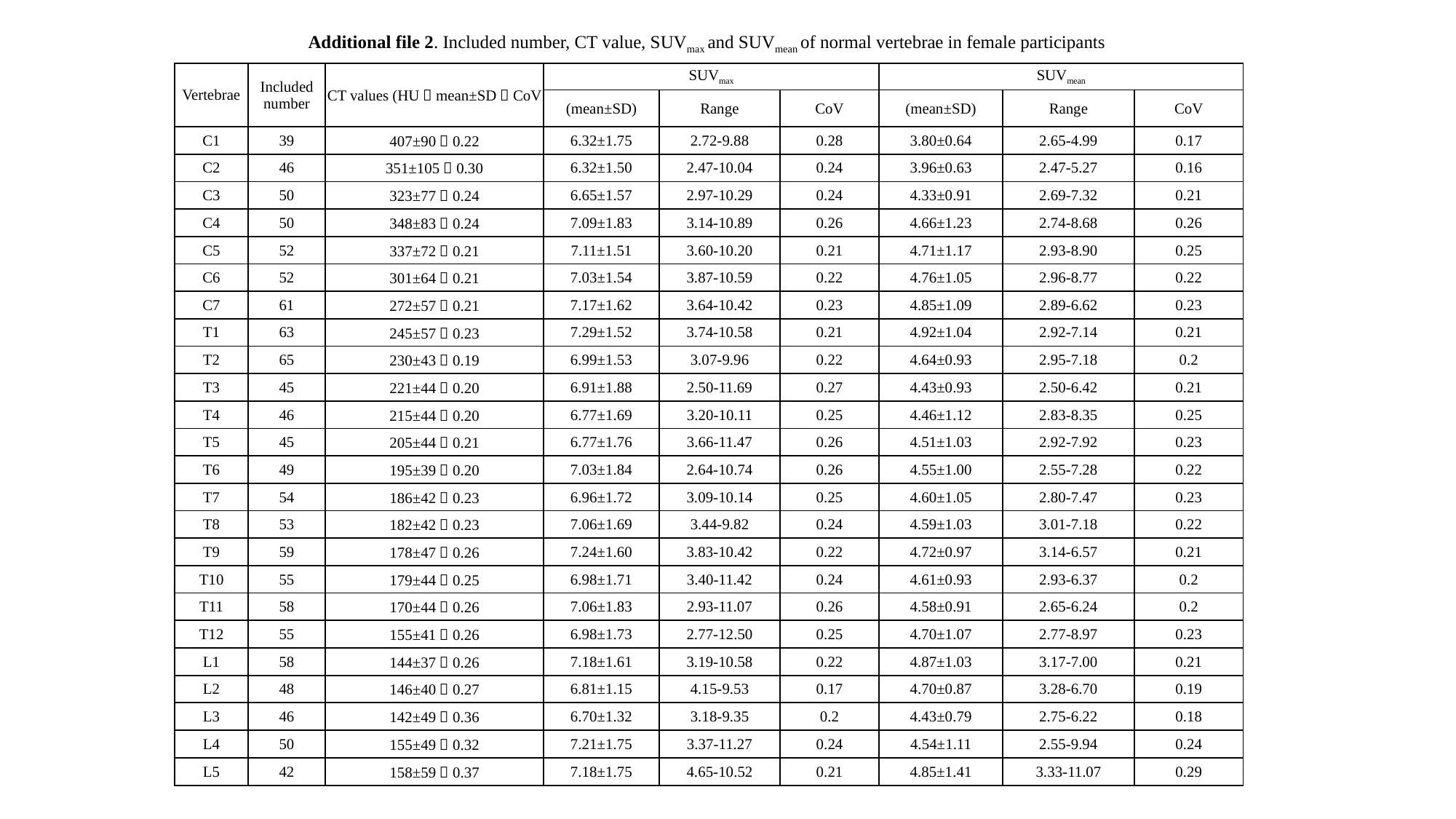

Additional file 2. Included number, CT value, SUVmax and SUVmean of normal vertebrae in female participants
| Vertebrae | Included number | CT values (HU）mean±SD，CoV | SUVmax | | | SUVmean | | |
| --- | --- | --- | --- | --- | --- | --- | --- | --- |
| | | | (mean±SD) | Range | CoV | (mean±SD) | Range | CoV |
| C1 | 39 | 407±90，0.22 | 6.32±1.75 | 2.72-9.88 | 0.28 | 3.80±0.64 | 2.65-4.99 | 0.17 |
| C2 | 46 | 351±105，0.30 | 6.32±1.50 | 2.47-10.04 | 0.24 | 3.96±0.63 | 2.47-5.27 | 0.16 |
| C3 | 50 | 323±77，0.24 | 6.65±1.57 | 2.97-10.29 | 0.24 | 4.33±0.91 | 2.69-7.32 | 0.21 |
| C4 | 50 | 348±83，0.24 | 7.09±1.83 | 3.14-10.89 | 0.26 | 4.66±1.23 | 2.74-8.68 | 0.26 |
| C5 | 52 | 337±72，0.21 | 7.11±1.51 | 3.60-10.20 | 0.21 | 4.71±1.17 | 2.93-8.90 | 0.25 |
| C6 | 52 | 301±64，0.21 | 7.03±1.54 | 3.87-10.59 | 0.22 | 4.76±1.05 | 2.96-8.77 | 0.22 |
| C7 | 61 | 272±57，0.21 | 7.17±1.62 | 3.64-10.42 | 0.23 | 4.85±1.09 | 2.89-6.62 | 0.23 |
| T1 | 63 | 245±57，0.23 | 7.29±1.52 | 3.74-10.58 | 0.21 | 4.92±1.04 | 2.92-7.14 | 0.21 |
| T2 | 65 | 230±43，0.19 | 6.99±1.53 | 3.07-9.96 | 0.22 | 4.64±0.93 | 2.95-7.18 | 0.2 |
| T3 | 45 | 221±44，0.20 | 6.91±1.88 | 2.50-11.69 | 0.27 | 4.43±0.93 | 2.50-6.42 | 0.21 |
| T4 | 46 | 215±44，0.20 | 6.77±1.69 | 3.20-10.11 | 0.25 | 4.46±1.12 | 2.83-8.35 | 0.25 |
| T5 | 45 | 205±44，0.21 | 6.77±1.76 | 3.66-11.47 | 0.26 | 4.51±1.03 | 2.92-7.92 | 0.23 |
| T6 | 49 | 195±39，0.20 | 7.03±1.84 | 2.64-10.74 | 0.26 | 4.55±1.00 | 2.55-7.28 | 0.22 |
| T7 | 54 | 186±42，0.23 | 6.96±1.72 | 3.09-10.14 | 0.25 | 4.60±1.05 | 2.80-7.47 | 0.23 |
| T8 | 53 | 182±42，0.23 | 7.06±1.69 | 3.44-9.82 | 0.24 | 4.59±1.03 | 3.01-7.18 | 0.22 |
| T9 | 59 | 178±47，0.26 | 7.24±1.60 | 3.83-10.42 | 0.22 | 4.72±0.97 | 3.14-6.57 | 0.21 |
| T10 | 55 | 179±44，0.25 | 6.98±1.71 | 3.40-11.42 | 0.24 | 4.61±0.93 | 2.93-6.37 | 0.2 |
| T11 | 58 | 170±44，0.26 | 7.06±1.83 | 2.93-11.07 | 0.26 | 4.58±0.91 | 2.65-6.24 | 0.2 |
| T12 | 55 | 155±41，0.26 | 6.98±1.73 | 2.77-12.50 | 0.25 | 4.70±1.07 | 2.77-8.97 | 0.23 |
| L1 | 58 | 144±37，0.26 | 7.18±1.61 | 3.19-10.58 | 0.22 | 4.87±1.03 | 3.17-7.00 | 0.21 |
| L2 | 48 | 146±40，0.27 | 6.81±1.15 | 4.15-9.53 | 0.17 | 4.70±0.87 | 3.28-6.70 | 0.19 |
| L3 | 46 | 142±49，0.36 | 6.70±1.32 | 3.18-9.35 | 0.2 | 4.43±0.79 | 2.75-6.22 | 0.18 |
| L4 | 50 | 155±49，0.32 | 7.21±1.75 | 3.37-11.27 | 0.24 | 4.54±1.11 | 2.55-9.94 | 0.24 |
| L5 | 42 | 158±59，0.37 | 7.18±1.75 | 4.65-10.52 | 0.21 | 4.85±1.41 | 3.33-11.07 | 0.29 |
