# Supplementary material for: Standardized uptake values of 99mTc-MDP in normal vertebrae assessed using quantitative SPECT/CT for differentiation diagnosis of benign and malignant bone lesions
Source: BMC Med Imaging. 2021 Feb 27;21:39. doi: 10.1186/s12880-021-00569-5 (PMC7913396; doi:10.1186/s12880-021-00569-5)
Supplement: Supplementary file 3 — Additional file 3. Differences of SUVmax and SUVmean between male and female participants in each vertebra. [file 12880_2021_569_MOESM3_ESM.pptx]

## Slide 1
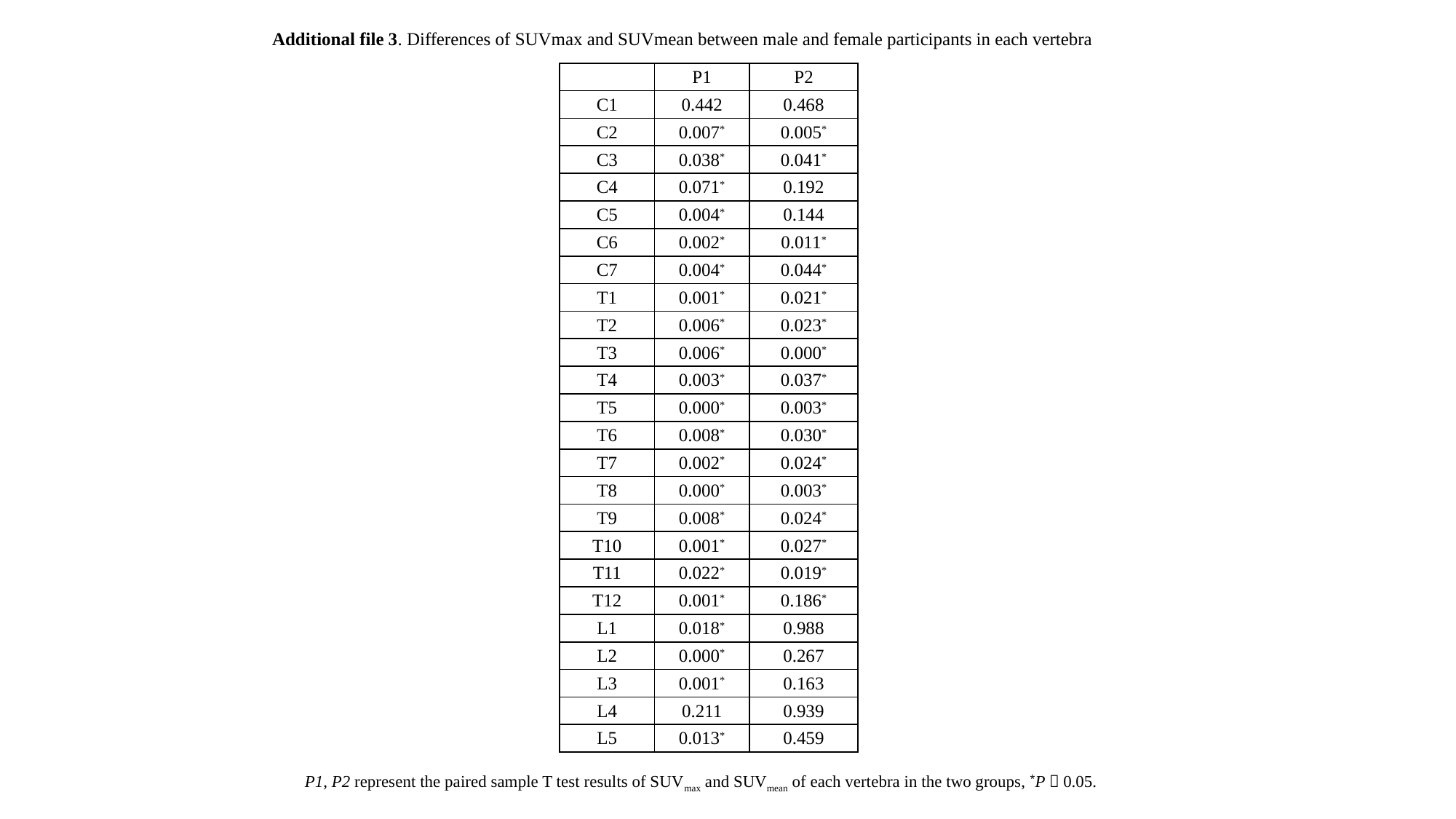

Additional file 3. Differences of SUVmax and SUVmean between male and female participants in each vertebra
| | P1 | P2 |
| --- | --- | --- |
| C1 | 0.442 | 0.468 |
| C2 | 0.007\* | 0.005\* |
| C3 | 0.038\* | 0.041\* |
| C4 | 0.071\* | 0.192 |
| C5 | 0.004\* | 0.144 |
| C6 | 0.002\* | 0.011\* |
| C7 | 0.004\* | 0.044\* |
| T1 | 0.001\* | 0.021\* |
| T2 | 0.006\* | 0.023\* |
| T3 | 0.006\* | 0.000\* |
| T4 | 0.003\* | 0.037\* |
| T5 | 0.000\* | 0.003\* |
| T6 | 0.008\* | 0.030\* |
| T7 | 0.002\* | 0.024\* |
| T8 | 0.000\* | 0.003\* |
| T9 | 0.008\* | 0.024\* |
| T10 | 0.001\* | 0.027\* |
| T11 | 0.022\* | 0.019\* |
| T12 | 0.001\* | 0.186\* |
| L1 | 0.018\* | 0.988 |
| L2 | 0.000\* | 0.267 |
| L3 | 0.001\* | 0.163 |
| L4 | 0.211 | 0.939 |
| L5 | 0.013\* | 0.459 |
P1, P2 represent the paired sample T test results of SUVmax and SUVmean of each vertebra in the two groups, *P＜0.05.
