# Supplementary figures and images for: Standardized uptake values of 99mTc-MDP in normal vertebrae assessed using quantitative SPECT/CT for differentiation diagnosis of benign and malignant bone lesions
Source: BMC Med Imaging. 2021 Feb 27;21:39. doi: 10.1186/s12880-021-00569-5 (PMC7913396; doi:10.1186/s12880-021-00569-5)

## Slide 1
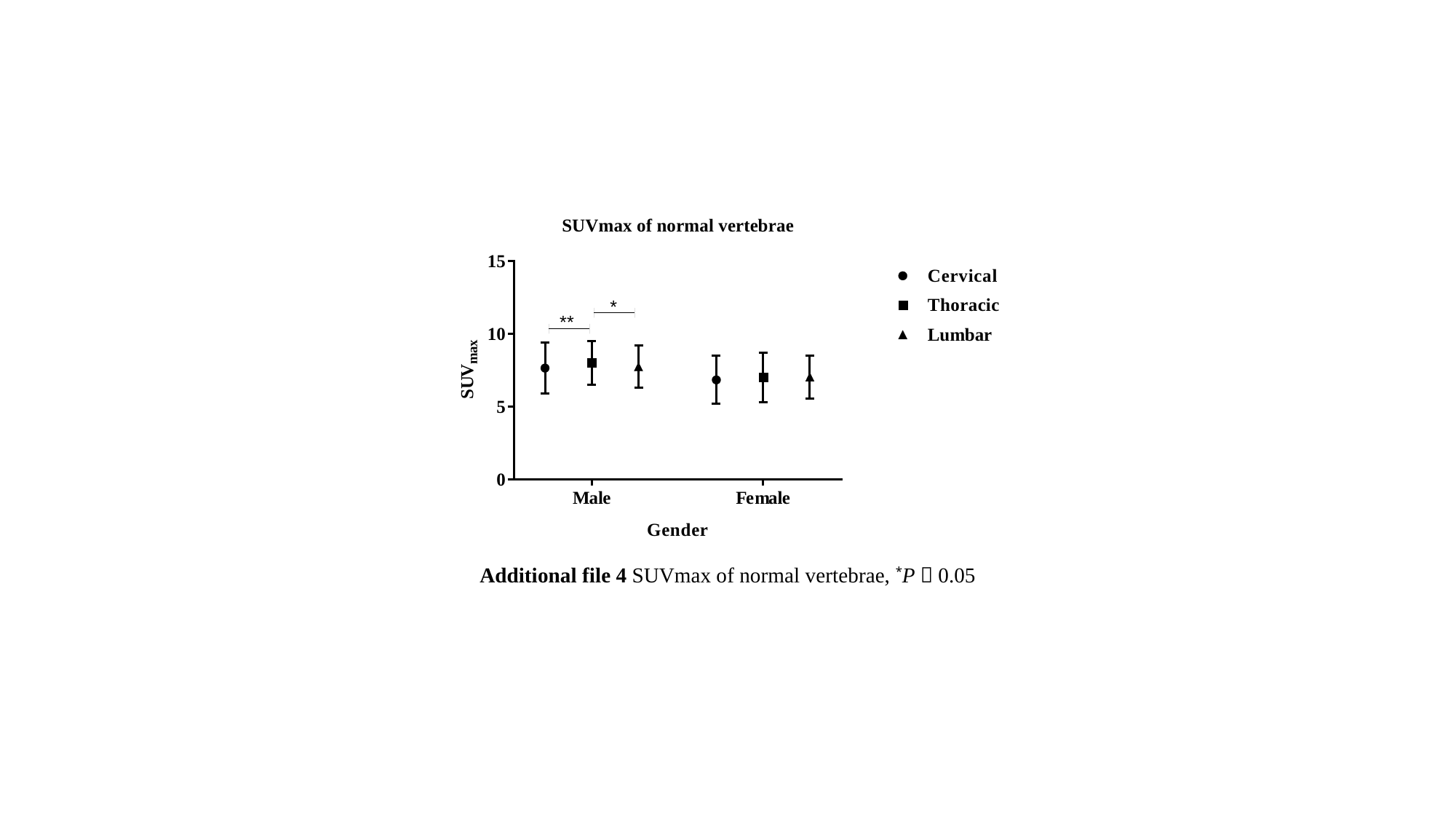

Additional file 4 SUVmax of normal vertebrae, *P＜0.05

Supplement: Supplementary file 4 — Additional file 4. SUVmax of normal vertebrae. [file 12880_2021_569_MOESM4_ESM.pptx]
